# Examining HIV Testing Coverage and Factors Influencing First-Time Testing Among Men Who Have Sex With Men in Zhejiang Province, China: Cross-Sectional Study Based on a Large Internet Survey

**DOI:** 10.2196/56906

**Published:** 2024-06-14

**Authors:** Lin He, Tingting Jiang, Wanjun Chen, Shaoqiang Jiang, Jinlei Zheng, Weiyong Chen, Hui Wang, Qiaoqin Ma, Chengliang Chai

**Affiliations:** 1 Zhejiang Provincial Center for Disease Control and Prevention Hangzhou China; 2 Coastal Service Center Hangzhou China

**Keywords:** HIV, testing, men who have sex with men, MSM, internet, pre-exposure prophylaxis, China, mobile phone

## Abstract

**Background:**

Men who have sex with men (MSM) constitute a significant population of patients infected with HIV. In recent years, several efforts have been made to promote HIV testing among MSM in China.

**Objective:**

This study aimed to assess HIV testing coverage and factors associated with first-time HIV testing among MSM to provide a scientific basis for achieving the goal of diagnosing 95% of patients infected with HIV by 2030.

**Methods:**

This cross-sectional study was conducted between July 2023 and December 2023. MSM were recruited from the “Sunshine Test,” an internet platform that uses location-based services to offer free HIV testing services to MSM by visiting the WeChat official account in Zhejiang Province, China. Participants were required to complete a questionnaire on their demographic characteristics, sexual behaviors, substance use, and HIV testing history. A logistic regression model was used to analyze first-time HIV testing and its associated factors.

**Results:**

A total of 7629 MSM participated in the study, with 87.1% (6647) having undergone HIV testing before and 12.9% (982) undergoing HIV testing for the first time. Multivariate logistic regression analysis revealed that first-time HIV testing was associated with younger age (adjusted odds ratio [aOR] 2.55, 95% CI 1.91-3.42), lower education (aOR 1.39, 95% CI 1.03-1.88), student status (aOR 1.35, 95% CI 1.04-1.75), low income (aOR 1.55, 95% CI 1.16-2.08), insertive anal sex role (aOR 1.28, 95% CI 1.05-1.56), bisexuality (aOR 1.69, 95% CI 1.40-2.03), fewer sex partners (aOR 1.44, 95% CI 1.13-1.83), use of rush poppers (aOR 2.06, 95% CI 1.70-2.49), unknown HIV status of sex partners (aOR 1.40, 95% CI 1.17-1.69), lack of awareness of HIV pre-exposure prophylaxis (aOR 1.39, 95% CI 1.03-1.88), and offline HIV testing uptake (aOR 2.08, 95% CI 1.80-2.41).

**Conclusions:**

A notable 12.9% (982/7629) of MSM had never undergone HIV testing before this large internet survey. We recommend enhancing HIV intervention and testing through internet-based platforms and gay apps to promote testing among MSM and achieve the target of diagnosing 95% of patients infected with HIV by 2030.

## Introduction

Men who have sex with men (MSM) constitute a significant demographic of patients infected with HIV in China. Due to widespread unprotected sexual behaviors, engagement with multiple sexual partners, and the use of recreational agents, HIV prevalence among MSM remains notably high [1]. The overall national prevalence of HIV among MSM in China from 2001 to 2018 was estimated to be 5.7% [2]. In China, the annual number of newly diagnosed HIV infections through homosexual transmission has increased from 2.5% in 2006 to 25.6% in 2022 [3]. The Joint United Nations Program on HIV/AIDS (UNAIDS) proposed a 95-95-95 target by 2030 [4]. The overall aim of the target was to achieve diagnosis of 95% of patients infected with HIV, 95% of diagnosed patients treated, and 95% of treated patients having achieved viral suppression to reduce HIV transmission, and ultimately end the HIV epidemic worldwide. In China, with the implementation of the “Four Frees and One Care” policy and lifelong follow-up care, achieving the second and third 95% targets is feasible. In 2022, a total of 92.8% of patients diagnosed with HIV received antiretroviral therapy, and 97% of treated patients achieved viral suppression (defined as HIV viral load <1000 copies/mL) in China [3]. The national estimate is that the proportion of patients infected with HIV was diagnosed at only 84% in 2022, and achieving a 95% diagnosis target for patients infected with HIV may be a huge challenge in China by 2030.

The “HIV testing as prevention” strategy stands as an effective approach to mitigate HIV transmission by encouraging early diagnosis of patients infected with the disease and linking them to antiretroviral therapy, thus reducing disease transmission [5,6]. There are 3 main HIV testing strategies among MSM in China. The first strategy is scaling up testing, expanding HIV testing coverage, and increasing testing frequency to prevent a substantial number of new HIV infections among MSM [7,8]. The second strategy is regular testing, which is a key prevention strategy for identifying and treating HIV infections among MSM [9,10]. The third strategy is HIV self-testing [11], which recommends self-testing as an innovative strategy and an additional testing approach to attain the UNAIDS targets to end HIV by 2030. With the implementation of the HIV testing strategy, annual HIV testing among MSM has reached nearly 600,000 in China [3], and an increasing number of MSM have received HIV interventions and are undergoing testing. To achieve the 95% diagnosis target for patients infected with HIV, promoting HIV testing among MSM is very important.

Previous studies found that younger individuals and students had lower HIV testing rates [9,12,13]. A higher HIV testing rate was observed in the developed country compared with the developing country. A meta-analysis study from the United States stated that only 15% of the MSM reported having first-time HIV testing [9], while the first-time HIV testing rate was 50.5% in Brazil [14] and 30.6% in Malaysia [15]. The first-time HIV testing rate in China was similar to other developing countries. A study showed that 30.1% and 29.4% of MSM underwent first-time HIV testing in Tianjin and Zhuhai, China, respectively [16]. A study on a large Chinese gay social media platform showed that 19.5% of MSM had never been tested for HIV [17]. An internet-distributed HIV self-testing study revealed that 16.6% of MSM had never been tested for HIV [18]. Some factors that have been associated with first-time HIV testing include Han ethnicity, HIV status, and income [19]. With the increasing popularity of the internet and smartphone usage in China, the internet penetration rate was expected to reach 76.4% in 2023 [20]. MSM who sought potential sexual partners from traditional places such as bars, parks, and baths, gradually transferred to internet-based dating apps, such as Blued app (BlueCity Group; a large gay Chinese social media platform). Consequently, the prevalence of gay app use among MSM has rapidly increased from 12.5% in 2015 to 52.6% in 2017 [21], suggesting that most MSM rely on gay apps to find sexual partners [22]. internet-based HIV interventions and testing [23], as well as gay apps [24], are considered simple ways of promoting HIV testing among MSM in China and increasing awareness of HIV status [25].

Zhejiang Province is located in eastern China, with an economically developed and prosperous internet economy. There are many companies that do e-commerce internet-based platforms, such as the Alibaba Group which was founded in Zhejiang Province and has had an HIV prevalence of 8% among MSM since 2008 [1]. Also, more than 40% of the annually newly diagnosed patients infected with HIV were MSM. Based on the developed internet economy, Zhejiang Province relies on the internet to conduct HIV intervention and testing among MSM, including internet-based consulting and mailing HIV testing kits for self-testing and using internet-based technology could increase the HIV self-testing rate among MSM [26]. Approximately 62,000 HIV testing cases occurred among MSM in Zhejiang Province in 2022, accounting for 10.6% of the national proportion [3], with over 70% of HIV testing in the province relying on the internet. Internet surveys offer broader coverage of the MSM population, rendering them more representative [27]. Identifying patients infected with HIV from the internet is key to diagnosing 95% of the infections by 2030. Therefore, this study aimed to assess HIV testing coverage based on a large internet survey and determine the factors associated with first-time HIV testing uptake among MSM in Zhejiang Province, China.

## Methods

### Sampling

A cross-sectional study based on a large internet survey was conducted between July 2023 and December 2023, targeting the MSM population.

### Study Participants

MSM participants were recruited based on inclusion criteria, which are (1) a minimum age of 16 years, (2) reported having sex with men in the past year, and (3) living in Zhejiang Province.

### Participant Recruitment and Data Collection

Sunshine Coast Public Welfare, a social organization, uses the internet to provide HIV interventions and testing services for the MSM population. The organization comprises 13 full-time social workers, 22 part-time social workers, and over 400 registered volunteers. Sunshine Coastal Public Welfare has established a digital HIV prevention service using location-based services. MSM populations in Zhejiang Province can apply for free HIV testing services by visiting the WeChat (Tencent Holdings Ltd, a very popular communication software in China) official account, “Sunshine Test.” Two options are available for MSM to access HIV testing services. First, they can opt to have the testing reagent mailed to them through courier and perform self-testing after receiving the reagent. Alternatively, they can choose offline services, where volunteers in their neighborhood provide HIV testing services, or they can visit nearby voluntary counseling and testing sites for MSM. During the study period, MSM had repeated HIV testing (more than 2 times); only the first testing record was retained in the study.

### Questionnaires

MSM who are applying for HIV testing services through “Sunshine Test” (a website by visiting WeChat in China) are required to complete a routine surveillance questionnaire. The questionnaire consists of 20 questions focusing on demographic characteristics such as age, marital status, education level, and sexual behavior characteristics such as sexual roles, number of sexual partners, whether to use rush poppers, sexual history, pre-exposure prophylaxis (PrEP), post-exposure prophylaxis (PEP), and HIV testing history. All questionnaires were completed on the “Sunshine Test.” MSM can apply for testing services after completing the questionnaire and being screened by a social worker or volunteer.

### Statistical Analysis

For descriptive analyses, categorical variables are presented as frequencies and proportions, while continuous variables are presented as medians and IQRs or means and SDs. The significance of the difference in the general demographic characteristics was tested by a chi-square test. Factors from the univariate analysis with *P*<.10 or those previously shown to be associated with the differences in sociodemographic characteristics among first-time HIV testing were included in multivariate logistic regression models, and adjusted odd ratios (aORs) were calculated along with corresponding 95% CIs. The statistical significance level was *P*≤.05 and β=.1. Statistical analyses were performed using SPSS (version 19.0; IBM Corp).

### Ethical Considerations

This study was approved by the Zhejiang Provincial Center for Disease Control and Prevention (2022-011-01). All participants provided written informed consent before the completion of the survey. Participants received free HIV testing and health counseling, and all study procedures were conducted in accordance with the approved guidelines and regulations.

## Results

**Figure 1:**
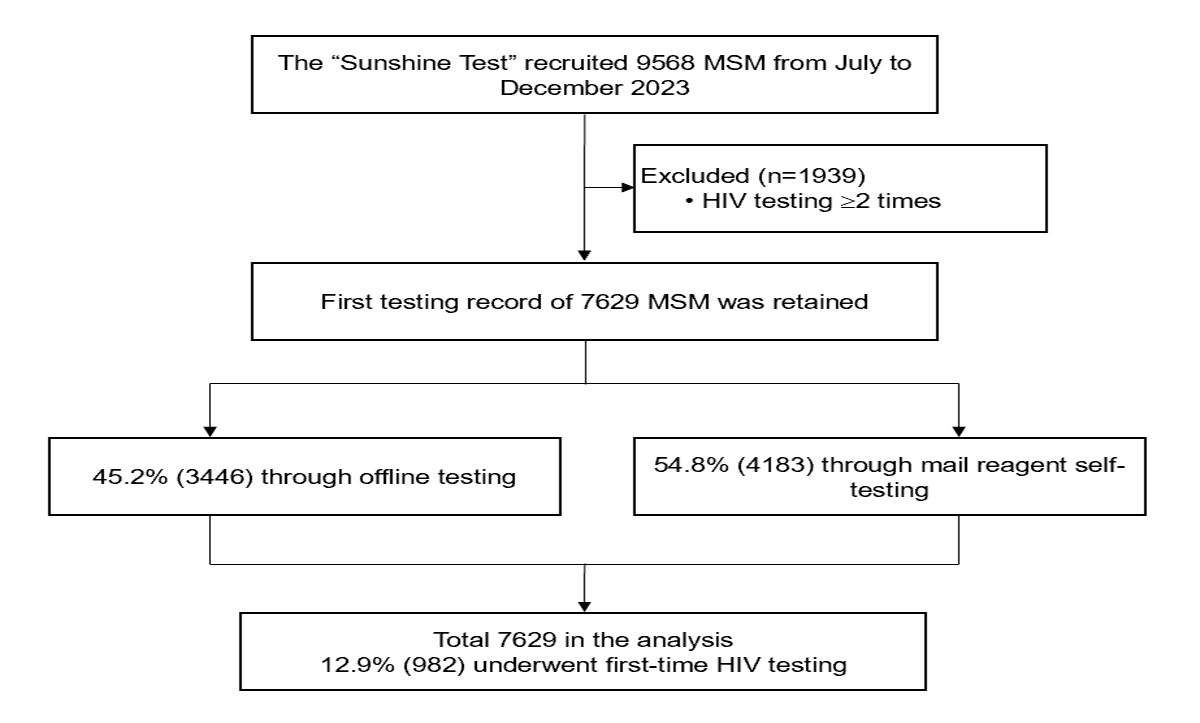
A cross-sectional study based on a large internet survey by visiting the official account of WeChat (a very popular communication software in China), “Sunshine Test.” A total of 7629 MSM participated in the study between July and December 2023 in Zhejiang Province, China. MSM: men who have sex with men.

A total of 7629 MSM participated in the study (Figure 1), with an average age of 29.0 (SD 8.5) years. Among them, 78.6% (5997/7629) were single, 50.7% (3869/7629) had a bachelor’s degree or higher, 16.4% (1254/7629) were students, and 49.1% (2985/7629) had a monthly income of less than US $700. The majority of 78.2% (5964/7629) had only male sexual partners, while 33.3% (2543/7629) had more than 2 sex partners in the past 3 months. Additionally, 31.2% (2380/7629) reported using rush poppers during sexual activity and 45.2% (3451/7629) were aware of their sex partner’s HIV status. The awareness rates of PrEP and PEP were 76.2% (5816/7629) and 78.1% (5960/7629), respectively. Furthermore, 54.8% (4183/7629) had HIV testing through mail reagent self-testing.

Of the 7629 participants, 12.9% (982/7629) underwent HIV testing for the first time. Factors associated with a high proportion of first-time HIV testing included being younger than 20 years old (30.9%, 133/430), having high school and below education (16.2%, 299/1851), being a student (21.6%, 271/1254), having a low monthly income (21.4%, 282/1320), identifying as bisexual (18.2%, 303/1665), having a small number of sex partners in the past 3 months (17.5%, 224/1277), frequently using rush poppers (18.5%, 198/1068), being unaware of their sex partners’ HIV status (18.6%, 275/1477), lacking awareness of HIV PrEP (18.9%, 342/1813), undergoing offline HIV testing (17.8%, 614/3446), and being HIV positive (24.4%, 20/82). The chi-square test showed that all factors except marital status, sex role, and history of sexually transmitted infections were associated with a high proportion of first-time HIV testing (Table 1).

Multivariate logistic regression analysis revealed that first-time HIV testing was associated with being younger than 20 years old (aOR 2.55, 95% CI 1.91-3.42), having attained high school education or lower (aOR 1.39, 95% CI 1.03-1.88), being a student (aOR 1.35, 95% CI 1.04-1.75), having a monthly income of less than US $350 (aOR 1.55, 95% CI 1.16-2.08), insertive anal sex role (aOR 1.28, 95% CI 1.05-1.56), identifying as bisexual (aOR 1.69, 95% CI 1.40-2.03), having a low number of sex partners in the past 3 months (aOR 1.44, 95% CI 1.13-1.83), frequently using rush poppers in the past 3 months (aOR 2.06, 95% CI 1.70-2.49), being unaware of their sex partners’ HIV status (aOR 1.40, 95% CI 1.17-1.69), lacking awareness of HIV PrEP (aOR 1.39, 95% CI 1.03-1.88), and undergoing offline HIV testing (aOR 2.08, 95% CI 1.80-2.41; Table 2).

**Table 1:** Social demographic and behavioral characteristics among men who have sex with men.

Variables		Values (N=7692), n (%)	First-time HIV testing (n=982), n (%)	chi-square (*df*)		*P* value	
**Age (years)^a^**					138.7 (2)		<.001
	<20	430 (5.6)	133 (30.9)				
	20-29	4526 (59.3)	568 (12.5)				
	≥30	2673 (35)	281 (10.5)				
**Marital status**					3.6 (2)		.16
	Single	5997 (78.6)	792 (13.2)				
	Married	1388 (18.2)	166 (12)				
	Divorced or separated	244 (3.2)	24 (9.8)				
**Education**					33.5 (3)		<.001
	High school and below	1851 (24.3)	299 (16.2)				
	College	1909 (25)	262 (13.7)				
	Bachelor’s degree	3145 (41.2)	335 (10.7)				
	Master’s degree or above	724 (9.5)	86 (11.9)				
**Occupation**					119.3 (3)		<.001
	Student	1254 (16.4)	271 (21.6)				
	Company employee	2843 (37.3)	265 (9.3)				
	Freelance	1022 (13.4)	117 (11.4)				
	Others	2510 (32.9)	329 (13.1)				
**Monthly income (US $)**					142.1 (3)		<.001
	<350	1320 (17.3)	282 (21.4)				
	350-599	1665 (21.8)	256 (15.4)				
	700-1399	3259 (42.7)	328 (10.1)				
	≥1400	1385 (18.2)	116 (8.4)				
**Sex roles**					5.4 (2)		.07
	Receptive anal sex	1750 (22.9)	197 (11.3)				
	Insertive anal sex	2652 (34.8)	349 (13.2)				
	Both	3227 (42.3)	436 (13.5)				
**Sex of partner**					53.9 (1)		<.001
	Male	5964 (78.2)	679 (11.4)				
	Male and female	1665 (21.8)	303 (18.2)				
**Number of sexual partners in the past 3 months**					68.6 (2)		<.001
	0	1277 (16.7)	224 (17.5)				
	1	3809 (49.9)	536 (14.1)				
	≥2	2543 (33.3)	222 (8.7)				
**Ever used rush poppers during sex behavior**					56.6 (2)		<.001
	Never use	5249 (68.8)	677 (12.9)				
	Occasional use	1312 (17.2)	107 (8.2)				
	Often use	1068 (14)	198 (18.5)				
**Awareness of partner’s HIV status**					59.4 (2)		<.001
	Yes	3451 (45.2)	427 (12.4)				
	Part	2701 (35.4)	280 (10.4)				
	No	1477 (19.4)	275 (18.6)				
**History of STI^b^**					<0.01 (1)		.98
	No	7428 (97.4)	956 (12.9)				
	Yes	201 (2.6)	26 (12.9)				
**Awareness of HIV PrEP^c^**					76.1 (1)		<.001
	No	1813 (23.8)	342 (18.9)				
	Yes	5816 (76.2)	640 (11)				
**Knowledge of HIV PEP^d^**					45.1 (1)		<.001
	No	1669 (21.9)	296 (17.7)				
	Yes	5960 (78.1)	686 (11.5)				
**HIV testing pathway**					137.1 (1)		<.001
	Offline testing	3446 (45.2)	614 (17.8)				
	Mail reagent self-testing	4183 (54.8)	368 (8.8)				
**HIV status**					9.8 (1)		<.002
	Positive	82 (1.1)	20 (24.4)				
	Negative	7547 (98.9)	962 (12.7)				

^a^Median 27 (IQR 23-33).

^b^STI: sexually transmitted infections.

^c^PrEP: pre-exposure prophylaxis.

^d^PEP: post-exposure prophylaxis.

**Table 2:** Factors associated with first-time HIV testing among men who have sex with men.

Variables		First-time HIV testing (n=982), n (%)	OR^a^ (95% CI)	*P* value	aOR^b^ (95% CI)	*P* value	
**Age (years;reference:≥**30**)**							
	<20	133 (30.9)	3.81 (3.00-4.84)	<.001	2.55 (1.91-3.42)	<.001	
	20-29	568 (12.5)	1.22 (1.05-1.42)	.01	1.38 (1.16-1.64)	<.001	
**Education (reference: master’s degree or above)**							
	High school and below	299 (16.2)	1.43 (1.11-1.85)	<.006	1.39 (1.03-1.88)	.03	
	College	262 (13.7)	1.18 (0.91-1.53)	.21	1.15 (0.86-1.54)	.33	
	Bachelor’s degree	335 (10.7)	0.88 (0.69-1.14)	.34	0.99 (0.75-1.29)	.93	
**Occupation** **(reference:** **o** **thers)**							
	Student	271 (21.6)	1.83 (1.53-2.18)	<.001	1.35 (1.04-1.75)	.02	
	Company employee	265 (9.3)	0.68 (0.57-0.81)	<.001	0.91 (0.76-1.11)	.36	
	Freelance	117 (11.4)	0.86 (0.69-1.07)	.18	0.85 (0.68-1.08)	.19	
**Monthly income (US $;** **reference: ≥US $1400)**							
	<$350	282 (21.4)	2.97 (2.36-3.74)	<.001	1.55 (1.16-2.08)	<.003	
	$350-$599	256 (15.4)	1.99 (1.58-2.51)	<.001	1.54 (1.20-1.99)	.001	
	$700-$1399	328 (10.1)	1.22 (0.98-1.53)	.07	1.05 (0.83-1.33)	.68	
**Sex roles** **(reference:** **receptive anal sex** **)**							
	Insertive anal sex	349 (13.2)	1.20 (1.00-1.44)	.06	1.28 (1.05-1.56)	.01	
	Both	436 (13.5)	1.23 (1.03-1.47)	.02	0.98 (0.80-1.19)	.82	
**Sex of partner** **(reference:** **m** **ale and female)**							
	Male and female	303 (18.2)	1.73 (1.49-2.00)	<.001	1.69 (1.40-2.03)	<.001	
**Number of sex partners in the past 3 months** **(reference: ≥2)**							
	0	224 (17.5)	2.22 (1.82-2.71)	<.001	1.44 (1.13-1.83)	<.003	
	1	536 (14.1)	1.71 (1.45-2.02)	<.001	1.68 (1.41-2.01)	<.001	
**Ever used rush poppers** **(reference:** **never use** **)**							
	Occasional use	107 (8.2)	0.60 (0.48-0.74)	<.001	0.85 (0.68-1.07)	.17	
	Often use	198 (18.5)	1.54 (1.29-1.83)	<.001	2.06 (1.70-2.49)	<.001	
**Awareness of partner’s HIV status** **(reference:** **yes** **)**							
	Part	280 (10.4)	0.82 (0.70-0.96)	.01	0.90 (0.76-1.06)	.22	
	No	275 (18.6)	1.62 (1.37-1.91)	<.001	1.40 (1.17-1.69)	<.001	
**Awareness of HIV PrEP^c^ (reference: no)**							
	No	342 (18.9)	1.88 (1.63-2.17)	<.001	1.56 (1.34-1.83)	<.001	
**HIV testing pathway** **(reference:** **offline testing** **)**							
	Offline testing	614 (17.8)	2.25 (1.96-2.58)	<.001	2.08 (1.80-2.41)	<.001	

^a^OR: odds ratio.

^b^aOR: adjusted odds ratio.

^c^PrEP: pre-exposure prophylaxis.

## Discussion

### Principal Findings

This study described HIV testing coverage based on a large internet survey and the factors associated with first-time HIV testing uptake among MSM in Zhejiang Province, China. Based on data from 7629 participants, we found that 87.1% (6647/7629) of MSM had undergone HIV testing, and 12.9% (982/7629) had never been tested before the survey. This 87.1% HIV testing coverage marks a significant increase from the 55.9% reported a decade ago (2013-2014) in Zhejiang Province [28]. Comparative analysis revealed that the HIV testing rate of 87.1% aligned with the 85% reported in a US meta-analysis study [9], surpassing rates found in Tianjin [16] and Zhuhai (70.6%) [19], as well as an internet-based study based on gay apps (80.5%) [17]. These findings underscore the need for continued efforts to expand HIV testing and increase testing coverage among MSM to achieve the target of diagnosing 95% of patients infected with HIV by 2030.

Consistent with previous studies, we found that younger individuals and students had lower HIV testing rates [9,12,13]. In China, nearly 3000 patients infected with HIV are diagnosed annually, more than 80% of whom are MSM [3]. An internet-based study found that younger MSM were probably deterred from seeking testing due to apprehension about health care or local office settings [13]. HIV molecular transmission clusters found students and nonstudents with HIV infections in the same transmission clusters [29]. However, the nonstudent MSM had an HIV prevalence of nearly 8%. Accordingly, a lack of awareness of the HIV epidemic in students among MSM may result in a low HIV testing rate. The internet and smartphones are essential tools for the student population, and a previous study showed that HIV intervention and testing through internet-based platforms or gay apps could increase HIV testing among younger MSM [24,30,31]. We suggest that strengthening awareness of the HIV epidemic among students and enhancing HIV testing education through internet-based platforms or gay apps will increase the HIV testing proportion of MSM students.

We found that participants with fewer sexual partners had low HIV testing rates. A study in China showed that individuals with 2 or more sexual partners were more likely to undergo HIV testing [32]. MSM with multiple sexual partners usually exhibit high-risk behaviors that prompt them to undergo HIV testing. Similarly, MSM who often used rush poppers had a higher HIV testing rate. Rush poppers are widely popular among MSM and have become the main substance type used during sexual activities [33]. Rush poppers prolong sexual activity and increase the rate of unprotected sex, thereby increasing their risk of HIV infection. Studies have shown that people who use rush poppers have more unprotected, casual sex, and increased HIV testing rates [9,34].

Moreover, we found that being unaware of a partner’s HIV status was associated with lower HIV testing rates. Serostatus disclosure strategies can reduce the risk of HIV infection and promote regular HIV testing which has been gradually promoted among MSM in China in recent years [35]. The serostatus disclosure strategy refers to knowing each other’s HIV status before any sexual activity. The strategy encourages using HIV status to decide whether to have sex or not. If both partners have the same HIV infection status, do not use condoms; and if the HIV infection status is inconsistent, use condoms [36]. Our study also showed that those who knew their sexual partners’ HIV status had a higher HIV testing rate. Therefore, it is recommended to continue implementing this strategy among MSM to increase HIV testing rates.

Participants who were aware of PrEP had higher rates of HIV testing. HIV PrEP and PEP are biological interventions that prevent HIV transmission, providing effective prevention opportunities for MSM at a high risk of HIV exposure, and can effectively reduce HIV infection and transmission [37]. In China, PEP guidelines were released by the National Center for Communicable Diseases Control in 2020. PEP has been widely promoted among MSM after 3 years of promotion. However, PrEP has only been piloted in some cities, and there was no national guideline for PrEP [3]. Therefore, in the results of this study, only those who had an awareness of PEP had a higher rate of HIV testing. Several studies have shown that unprotected sex and sexually transmitted infections continue to increase among MSM who have used PrEP drugs [38]. Previous studies have also shown that the PrEP project effectively promoted HIV testing among MSM [39], and greater odds of PrEP awareness were associated with HIV testing [40].

This study had some limitations. First, the study recruited MSM who used web-based apps for HIV testing, 88.5% (6753/7629) of whom were younger than 40 years. It did not capture a few MSM who came from traditional places such as bars and baths, especially those who were older than 50 years. Previous studies have shown that this population has a higher HIV infection rate and a lower HIV testing rate [32]. Therefore, the results of this study apply only to MSM who rely on the internet for HIV testing. Second, participants who opted for HIV self-testing through mailed reagents may introduce bias, as the feedback on true HIV-positive results could be limited. Finally, the use of routine surveillance questionnaires may not capture all factors associated with HIV testing as identified in previous studies.

### Conclusions

A significant finding from the large internet survey was that 12.9% (982/7629) of MSM had never undergone HIV testing before the study. We suggest enhancing HIV intervention and testing through internet-based platforms and gay apps to promote HIV testing among MSM. Future studies could explore strategies to increase HIV testing rates among underrepresented demographics, such as older MSM and those who do not rely on web-based testing services, ultimately helping to meet the target of diagnosing 95% of patients infected with HIV by 2030.

## Abbreviations

aOR: adjusted odds ratio
MSM: men who have sex with men
PEP: post-exposure prophylaxis
PrEP: pre-exposure prophylaxis
UNAIDS: Joint United Nations Program on HIV/AIDS

## Data Availability

The data sets generated and analyzed during this study are available from the corresponding author on reasonable request.
